# Endoscopic third ventriculostomy for idiopathic normal pressure hydrocephalus: association of cerebrospinal fluid flow dynamics with outcome

**DOI:** 10.1016/j.bas.2026.107452

**Published:** 2026-09-30

**Authors:** Karel Pištěk, Martin Sameš, Daniel Adámek, Tomáš Radovnický

**Affiliations:** a Second Faculty of Medicine, Charles University, Prague, Czech Republic; b Neurosurgical Department, J.E. Purkynje University, Masaryk Hospital Krajská Zdravotní a.s., Ústí nad Labem, Czech Republic; c Radiological Department, J.E. Purkynje University, Masaryk Hospital Krajská Zdravotní a.s., Ústí nad Labem, Czech Republic

**Keywords:** Idiopathic normal pressure hydrocephalus, Endoscopic third ventriculostomy, Phase-contrast MRI, Cerebrospinal fluid dynamics, Aqueductal stroke volume, Ventriculoperitoneal shunt

## Abstract

**Introduction:**

Endoscopic third ventriculostomy (ETV) has been proposed for selected patients with idiopathic normal pressure hydrocephalus (iNPH), but predictors of sustained benefit are uncertain. Phase-contrast MRI (PC-MRI) may quantify cerebrospinal fluid (CSF) pulsatility and support patient selection.

**Research question:**

Is sustained clinical improvement after primary ETV associated with preoperative aqueductal CSF flow parameters measured by PC-MRI?

**Material and methods:**

In this prospective single-centre observational study, patients with iNPH underwent primary ETV after standardized diagnostic work-up. Preoperative assessment included the Kiefer scale and PC-MRI measurements at the cerebral aqueduct: peak velocity (PV), aqueductal stroke volume (ASV), and net ASV. Clinical follow-up was performed for 12 months. Sustained response was defined as an NPH recovery rate ≥3 at 3 and 12 months without secondary ventriculoperitoneal shunt implantation. Analyses were exploratory.

**Results:**

Thirty-one patients were included, of whom 10 (32.3%) achieved sustained clinical improvement. Responders demonstrated significantly higher preoperative ASV values compared to non-responders. Among the evaluated parameters, ASV was the only variable significantly associated with clinical response. Receiver operating characteristic (ROC) analysis demonstrated good discriminatory capacity for ASV (area under the curve [AUC] 0.83). An exploratory cut-off value of 280 μl yielded a sensitivity of 90%, specificity of 71%, positive predictive value of 60%, and negative predictive value of 94%.

**Discussion and conclusion:**

Higher preoperative ASV was associated with sustained clinical improvement after primary ETV in this small iNPH cohort. PC-MRI-derived ASV may help identify candidates unlikely to benefit from ETV, but the proposed threshold requires external validation before clinical use.

## Highlights

• Higher aqueductal stroke volume was associated with sustained response.
• An exploratory 280-μl cutoff showed 90% sensitivity and 71% specificity.
• Endoscopic third ventriculostomy produced sustained 12-month improvement in 32.3%.
• Peak aqueductal velocity decreased after surgery but did not predict response.
• Secondary shunting improved 80% after unsuccessful third ventriculostomy.

## Graphical abstract

**Figure:**
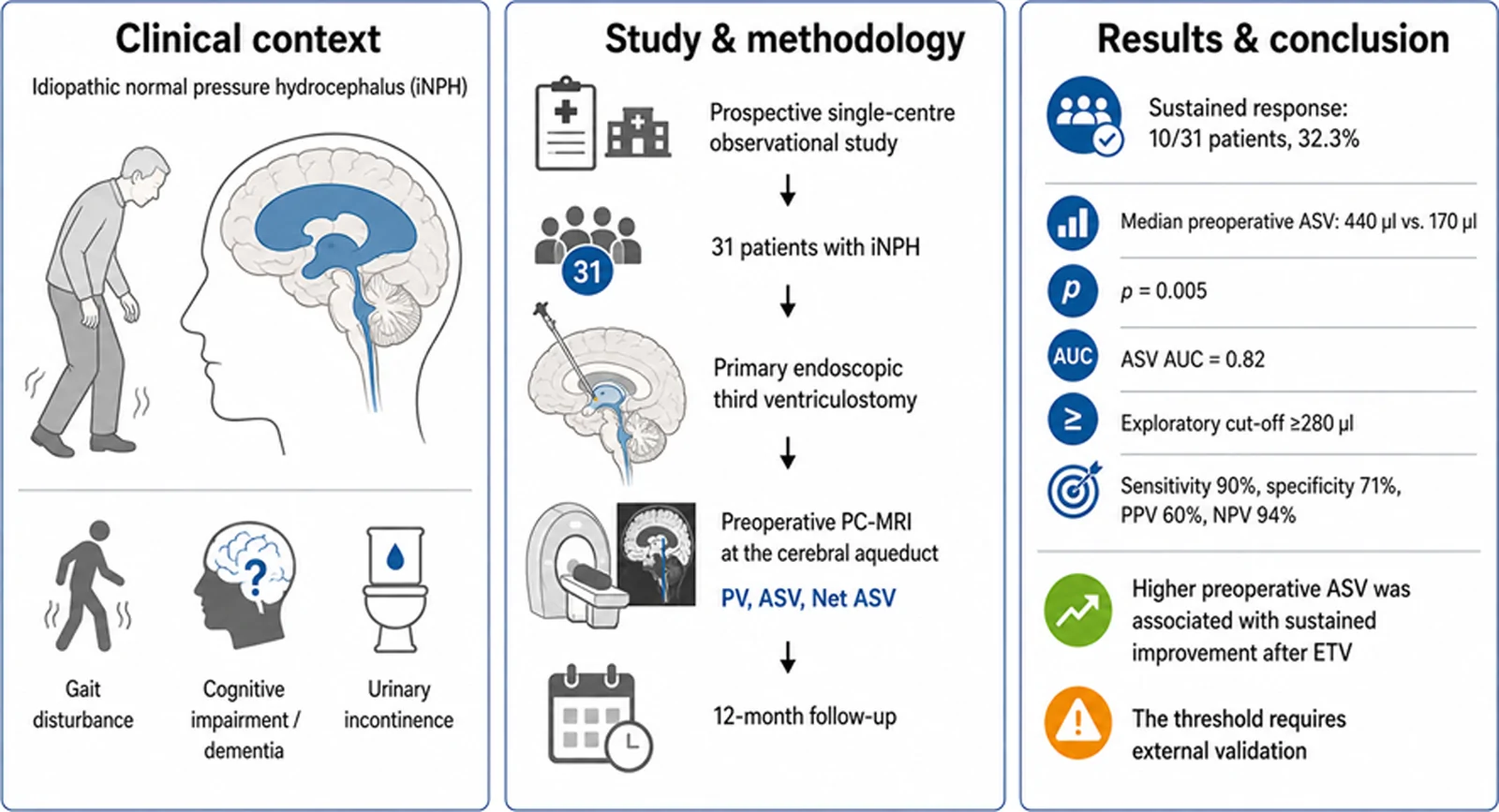

## 1 Introduction

Idiopathic normal pressure hydrocephalus (iNPH) is a potentially treatable neurological disorder, originally described by Hakim and Adams in 1965, characterized by gait disturbance, cognitive impairment, urinary dysfunction, and ventriculomegaly (Adams et al., 1965; Hakim and Adams, 1965). It is typically associated with cerebrospinal fluid (CSF) opening pressure within the guidelinebased range for iNPH, commonly reported as 5–18 mmHg, equivalent to 7–24.5 cmH_2_O, when measured by lumbar puncture in the horizontal lateral decubitus position (Norager et al., 2021; Relkin et al., 2005). NPH is regarded as a form of chronic communicating hydrocephalus and is classified as idiopathic or secondary. In contrast to secondary NPH (sNPH), which occurs after identifiable intracranial events such as subarachnoid hemorrhage, meningitis, traumatic brain injury, stroke, or intracranial tumor, iNPH develops in the absence of an identifiable cause (Daou et al., 2016; Marmarou et al., 2005). The present study focuses exclusively on patients with iNPH.

Ventriculoperitoneal (VP) shunting remains the standard surgical treatment for iNPH, with reported clinical improvement in approximately 75% of appropriately selected patients (Reddy et al., 2014; Giordan et al., 2018). However, shunt therapy is associated with clinically relevant complication and revision rates, including infection, overdrainage, mechanical malfunction, and long-term device dependence (Reddy et al., 2014; Giordan et al., 2018; Prusseit et al., 2009). In an effort to reduce shunt-related morbidity, endoscopic third ventriculostomy (ETV) has been explored as a potential alternative in selected patients with iNPH. While ETV is an established treatment for obstructive hydrocephalus, its role in iNPH remains controversial and it is not an established standard treatment. Reported outcomes are variable, and appropriate patient selection remains critical (Tasiou et al., 2016).

Prospective data evaluating the relationship between preoperative CSF flow dynamics and sustained clinical response after primary ETV in patients with iNPH remain limited. A better understanding of this association may help refine patient selection and support more individualized surgical decision-making. The aim of this prospective, single-center, nonrandomized observational study was to evaluate clinical outcome after primary ETV in patients with iNPH and to determine whether preoperative phase-contrast magnetic resonance imaging (PC-MRI)-derived CSF flow parameters are associated with sustained clinical response. We hypothesized that higher preoperative aqueductal CSF pulsatility would be associated with favorable clinical outcome after ETV.

## 2 Methods

### 2.1 Patient selection

The study was designed as a single-institution, prospective, non-randomized observational study. The study protocol, including predefined variables, clinical outcomes, and follow-up schedule, was established prior to patient enrollment. The study was conducted between March 2021 and January 2025.

The primary objective was to evaluate the association between CSF flow dynamics measured by PC-MRI and clinical response following primary ETV in patients with iNPH (ICD-10: G91.2).

All consecutive patients who fulfilled the diagnostic eligibility criteria and underwent primary ETV for iNPH at the study institution were included during the recruitment period.

Patients fulfilling diagnostic criteria for iNPH were offered, after standardized counseling, a choice between primary ETV and VP shunt implantation. Treatment allocation was not randomized and was based on patient and family preference following informed decision-making, representing a potential source of selection bias. Only patients undergoing primary ETV were included in the present analysis.

Inclusion and exclusion criteria were applied prior to enrollment and were based on internationally accepted diagnostic criteria for iNPH published in 2005 (Relkin et al., 2005).

#### 2.1.1 Inclusion criteria

• Ventriculomegaly involving three or four ventricles on CT or MRI, defined as Evans' index ≥ 0.3 (Evans, 1942; Nakajima et al., 2021).
• Presence of at least one symptom of the Hakim–Adams triad (gait disturbance, cognitive impairment, urinary and/or fecal incontinence).
• Positive result of at least one supplementary diagnostic test:
- CSF tap test showing ≥10% improvement in gait time 5 h after removal of 50 ml of CSF; or
- LIT demonstrating increased resistance of CSF outflow (Rout), defined as Rout ≥ 11 mmHg/ml/min (Albeck et al., 1991; Gallagher et al., 2019; Kim et al., 2015).

#### 2.1.2 Exclusion criteria

• History of major intracranial pathology, including subarachnoid hemorrhage, meningitis, traumatic brain injury, stroke, or intracranial tumor (Daou et al., 2016).
• Macrocephaly defined as head circumference > 59 cm in adult males or > 57.5 cm in adult females (Oi et al., 2000).
• Enlargement or destruction of the sella turcica on MRI (Oi et al., 2000).
• Macroscopic obstruction of the ventricular system.
• Aqueductal stenosis or occlusion visible on sagittal MRI (Palandri et al., 2022).
• Opening pressure measured by lumbar puncture in the lateral decubitus position exceeding 18 mmHg (24.5 cmH_2_O) (Relkin et al., 2005).
• Presence of papilledema on fundoscopy.
• Clinically relevant improvement of motor symptoms after L-DOPA administration, suggesting an alternative diagnosis such as Parkinson’s disease or another parkinsonian syndrome.

Post-treatment deaths and incomplete follow-up were reported separately in the participant flow and were not considered pre-treatment eligibility criteria.

Patients who preferred primary VP shunt implantation after counseling were not included in the present ETV cohort.

The study was approved by the local ethics committee, and all patients provided written informed consent.

This observational cohort study is reported in accordance with the STROBE statement (Vandenbroucke et al., 2007).

### 2.2 Preoperative assessment

For all eligible patients, baseline demographic data (age, sex) were recorded. Preoperative clinical status was standardized using the Kiefer scale (preOP) (Kiefer et al., 2003, 2006). The total score was calculated as the sum of subscores evaluating cognitive function (M preOP), gait disturbance (G preOP), continence impairment (I preOP), headache (H preOP), and dizziness (D preOP).

One day prior to surgery, all patients underwent brain MRI according to a standardized protocol including PC-MRI sequences, 3D CSF DRIVE, axial T2-weighted imaging (T2WI), and coronal T1-weighted imaging (T1WI).

Based on preoperative MRI, the following morphological and physiological parameters were systematically evaluated.

- NPH Radscale (Kockum et al., 2018)
- Presence of disproportionately enlarged subarachnoid space hydrocephalus (DESH), assessed on coronal images (Ishikawa et al., 2016)
- Bowing of the third ventricle, assessed on sagittal images (Dlouhy et al., 2012)
- Patency of the cerebral aqueduct on 3D CSF DRIVE sequences (sagittal plane)
- Preoperative peak velocity (PV preOP) (Whitley et al., 2024)
- Preoperative aqueductal stroke volume (ASV preOP) (Whitley et al., 2024)
- Preoperative net ASV (Net ASV preOP) (Whitley et al., 2024)

### 2.3 MRI protocol

MRI examinations were performed using a 3T Philips Ingenia® scanner equipped with a 20-channel head and neck phased-array coil (Philips Medical Systems®). Standard morphological sequences included axial T2WI (slice thickness 4 mm), coronal T1WI (4 mm), and sagittal 3D CSF DRIVE sequence with isotropic resolution (1 mm).

PC-MRI was subsequently performed using a predefined CSF Quantitative Flow (QF) protocol within the Phase Processing Unit (PPU) module. Measurements were obtained in a plane perpendicular to the cerebral aqueduct. Acquisition parameters were: repetition time (TR) 12.272 ms, echo time (TE) 8.062 ms, flip angle 15°, voxel size 0.59 × 0.84 × 4 mm, slice thickness 4 mm, reconstructed voxel size 0.59 × 0.59 × 4 mm, and reconstruction matrix 256 × 179. Total acquisition time was approximately 6 min. The sequence was synchronized with the cardiac cycle using peripheral pulse oximetry.

All PC-MRI sequences were acquired with a fixed velocity encoding (VENC) value according to the manufacturer’s preset protocol, identical for all examinations. Data were acquired in a single slice perpendicular to the aqueduct at the mid-tectal level, with the aqueduct centered within the region of interest. Images were reconstructed across multiple cardiac phases.

CSF flow parameters (PV, ASV, Net ASV) were automatically extracted using the vendor-provided software.

ASV was defined as the absolute sum of the craniocaudal and caudocranial CSF volumes passing through the mesencephalic aqueduct during one cardiac cycle (μl/cycle).

All preoperative and postoperative MRI examinations were evaluated by a single neuroradiologist blinded to clinical outcomes.

### 2.4 Endoscopic third ventriculostomy

All procedures were performed under general anesthesia using a standardized technique. A right frontal burr hole was placed at the level of the coronal suture approximately 3 cm lateral to the midline. Following dural opening, an introducer was inserted into the central portion of the right lateral ventricle.

A rigid neuroendoscope - PaediScope® (outer diameter 3 mm) was advanced through the introducer into the ventricular system. After inspection of the right lateral ventricle, the right foramen of Monro was identified, and the endoscope was advanced into the third ventricle for visualization of its floor.

A perforation was created in the tuber cinereum between the mammillary bodies using monopolar coagulation and subsequently dilated with a double-balloon Fogarty catheter. After stoma creation, the interpeduncular cistern was inspected, and any arachnoid adhesions or Liliequist’s membrane were gently fenestrated when present. Adequate stoma patency was confirmed by direct endoscopic visualization and observation of third ventricular floor pulsations.

Following completion of the procedure, the endoscope and introducer were removed.

The dural opening was sealed with hemostatic material, and the burr hole was covered with a small titanium plate.

All procedures were performed by two neurosurgeons experienced in neuroendoscopic surgery.

### 2.5 Postoperative follow-up

Follow-up MRI using the same protocol was scheduled within one month after ETV. All 31 surviving participants completed postoperative MRI assessment. Postoperative assessment included PV postOP, ASV postOP, and Net ASV postOP. Patency of the stoma was evaluated on 3D CSF DRIVE sequences by assessing for flow void at the third ventricular floor.

Clinical follow-up was conducted at 3, 6, 9, and 12 months postoperatively. At each visit, clinical status was assessed using the Kiefer scale. The NPH recovery rate was calculated at 3 and 12 months to evaluate clinical response to ETV (Meier, 2002). Follow-up visits were in-person outpatient assessments performed by two dedicated study neurosurgeons. Clinical status and any complications, deterioration, or improvement were systematically recorded in the medical record.

### 2.6 Assessment of ETV effect

The primary clinical outcome was response to ETV. Clinical effect was predefined and assessed in a binary manner based on the NPH recovery rate. Primary evaluation (Effect 1/0) was performed at 3 months postoperatively. ETV was considered effective (Effect 1) if the NPH recovery rate was ≥ 3. Otherwise, it was classified as ineffective (Effect 0), and secondary VP shunt implantation was indicated.

At the 6- and 9-month visits, the NPH recovery rate was not calculated. An unchanged or increased total Kiefer score compared with the preoperative assessment was considered evidence of absent clinical benefit or clinical deterioration and prompted consideration of secondary VP shunt implantation. Sustained responders were defined as patients with Effect 1 at 3 months who maintained this status at 12 months without requiring VP shunt implantation.

Secondary outcomes included postoperative changes in CSF flow parameters and descriptive analysis of patients requiring secondary VP shunting. Associations between preoperative CSF flow parameters and clinical outcome were evaluated statistically.

### 2.7 Statistical analysis

Statistical analysis was exploratory and aimed to identify potential associations between ETV outcome and evaluated variables. Variables included demographic data (age, sex), morphological findings (DESH, NPH Radscale), clinical status (total and subcomponent Kiefer scores), and CSF flow parameters (PV, ASV, Net ASV).

Normality of continuous variables was assessed using the Shapiro-Wilk test. Paired comparisons were performed using paired *t*-test or Wilcoxon signed-rank test as appropriate.

Comparisons between Effect 1 and Effect 0 groups were performed using the Mann-Whitney *U* test. Categorical variables were analyzed using chi-square or Fisher’s exact test.

Associations were evaluated using Spearman correlation analysis. Variables with p < 0.10 in univariable analysis were included in exploratory multivariable logistic regression.

Results were reported as odds ratios (OR) with 95% confidence intervals.

Discriminatory performance of selected CSF flow parameters was assessed using receiver operating characteristic (ROC) analysis (area under the curve, AUC). Optimal cut-off values were determined using the Youden index and interpreted as hypothesis-generating.

Sensitivity, specificity, positive predictive value, and negative predictive value were calculated.

Given the limited sample size, no a priori power calculation was performed. Statistical analyses were conducted using Python (version 3.11). Statistical significance was set at p < 0.05.

Additionally, a descriptive analysis was performed for the subgroup of patients undergoing secondary VP shunt implantation. Associations between preoperative parameters and clinical response to secondary treatment were evaluated using Spearman correlation.

### 2.8 Study size

No formal a priori sample-size calculation, prespecified sample-size target, or formal stopping rule was used. All consecutive eligible patients who underwent primary ETV were included between March 2021 and January 2025. Recruitment ended in January 2025 after the study grant was exhausted, this decision was made without knowledge of interim clinical outcomes or PC-MRI results. Given the small cohort and 10 sustained response events, regression and ROC analyses were exploratory.

## 3 Results

Between March 2021 and January 2025, 42 patients were diagnosed with iNPH and indicated for neurosurgical treatment. Primary ETV was chosen by 36 patients, whereas 6 patients preferred primary VP shunt implantation. Based on preoperative MRI (3D CSF DRIVE sequence), two patients were excluded due to aqueductal stenosis and were subsequently managed under the diagnosis of late-onset idiopathic aqueductal stenosis (LIAS) (Palandri et al., 2022).

A total of 34 patients underwent ETV. Imaging features consistent with DESH were present in 12 patients (35.3%), and bowing of the third ventricular floor was observed in one patient. Intraoperatively, pulsations of the stoma at the floor of the third ventricle were observed in all patients following ETV. There were no procedure-related hemorrhages, wound or intracranial infections, cerebrospinal fluid leakage, new postoperative neurological deficits, clinically relevant intraventricular or subdural bleeding, or ETV-related reoperations. No procedure-related mortality occurred. Three patients died before postoperative MRI and initial clinical follow-up; two deaths were due to pneumonia and one to urinary tract infection in the context of general frailty. All three deaths were judged unrelated to the surgical procedure.

The final analysis included 31 patients who underwent primary ETV for iNPH. All 31 patients completed postoperative imaging and clinical follow-up. The patient selection, exclusions, treatment allocation, and follow-up are summarized in the STROBE flow diagram (Fig. 1). (Vandenbroucke et al., 2007)

**Fig. 1:**
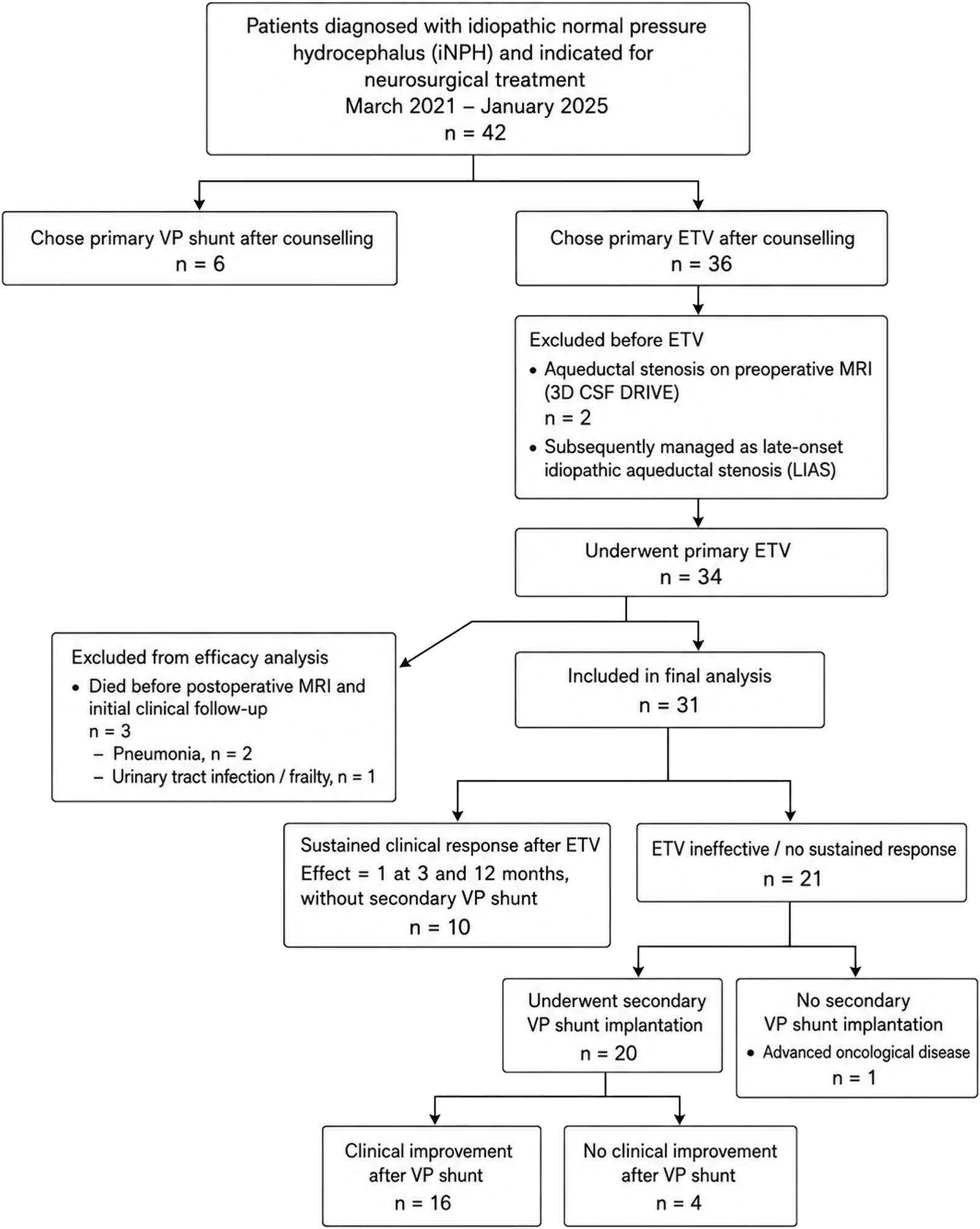
STROBE flow diagram of patient selection, treatment allocation, follow-up, and clinical outcoms.

The mean age was 74.5 years (median 73 years). The cohort comprised 19 men (61%) and 12 women (39%). Sustained clinical response (Effect = 1 at both 3 and 12 months without subsequent VP shunt implantation) was achieved in 10 patients (32.3%).

In the overall cohort, ETV was associated with a statistically significant reduction in PV (median 8.6 → 7.35 cm/s; p = 0.039). ASV decreased only modestly following surgery, and this change did not reach statistical significance (median 260 → 225 μl; p = 0.379).

Postoperative changes in PV, ASV, and Net ASV did not differ significantly between patients with and without sustained clinical response (all p > 0.1). Baseline demographic, clinical, radiological, and PC-MRI characteristics according to sustained ETV response are summarized in Table 1. Postoperative clinical and PC-MRI characteristics according to sustained ETV response are summarized in Table 2.

**Table 1:** Baseline characteristics according to sustained ETV response.

Variable	Overall (n = 31)	ETV non-responders (n = 21)	Sustained ETV responders (n = 10)	p value
Age, years	73 (71–79.5)	73 (71–80)	74 (71–79)	0.983
Male sex	19/31 (61.3%)	13/21 (61.9%)	6/10 (60.0%)	1.000
NPH Radscale	9 (7–9.5)	9 (7–10)	8 (7–9)	0.607
DESH present	10/31 (32.3%)	7/21 (33.3%)	3/10 (30.0%)	1.000
PV (preOP), cm/s	8.6 (6.3–11.1)	7.7 (5.2–10.2)	10.9 (8.6–11.2)	0.087
ASV (preOP), μl	260 (125–385)	170 (100–310)	440 (350–525)	0.005
Net ASV (preOP), μl	20 (10–40)	10 (10–30)	45 (22–75)	0.009
Kiefer score (preOP)	9 (6–12.5)	9 (7–14)	6.5 (4–9.5)	0.042
Mental state (preOP)	1 (1–4)	4 (1–4)	1 (1–1)	0.036
Gait disorders (preOP)	4 (2–5)	4 (2–5)	2 (2–4.8)	0.216
Incontinence (preOP)	3 (1–4)	3 (3–4)	3 (0.2–3.8)	0.272
Headache (preOP)	0 (0–0)	0 (0–0)	0 (0–0)	1.000
Dizziness (preOP)	0 (0–1)	0 (0–1)	0 (0–0.8)	0.876

**Table 2:** Postoperative clinical and PC-MRI characteristics according to sustained ETV response.

Variable	Overall (n = 31)	ETV non-responders (n = 21)	Sustained ETV responders (n = 10)	p value
PV (postOP), cm/s	7.3 (5.3–10.7)	6.7 (4.8–10.3)	8.8 (6–10.7)	0.403
ASV (postOP), μl	225 (115–387.5)	195 (100–272.5)	410 (310–450)	0.013
Kiefer score (postOP)	6 (3.5–9.5)	8 (5–12)	3 (2.8–4.5)	0.006
NPH recovery rate (3 months)	1.7 (0–4.5)	1.4 (0–3.6)	4.8 (3.5–5.3)	0.025
NPH recovery rate (12 months)	3.2 (1.2–5.6)	0 (0–1)	5 (3.5–7.5)	0.003
Mental state (postOP)	1 (0.5–2.5)	1 (1–4)	0 (0–1)	0.004
Gait disorders (postOP)	2 (2–4)	4 (2–4.5)	2 (0–2)	0.015
Incontinence (postOP)	3 (1–4)	3 (2–4)	1 (0.8–3)	0.068
Headache (postOP)	0 (0–0)	0 (0–0)	0 (0–0)	0.922
Dizziness (postOP)	0 (0–0)	0 (0–0)	0 (0–0)	0.863

Patients with sustained clinical response demonstrated significantly higher preoperative ASV values (median 440 μl vs. 170 μl; p = 0.0049) and net ASV values (median 45 μl vs. 10 μl; p = 0.0092) compared to non-responders (Fig. 2). A trend toward higher preoperative PV values was observed among responders (10.9 vs. 7.7 cm/s), although this difference did not reach statistical significance (p = 0.087) (Fig. 3).

**Fig. 2:**
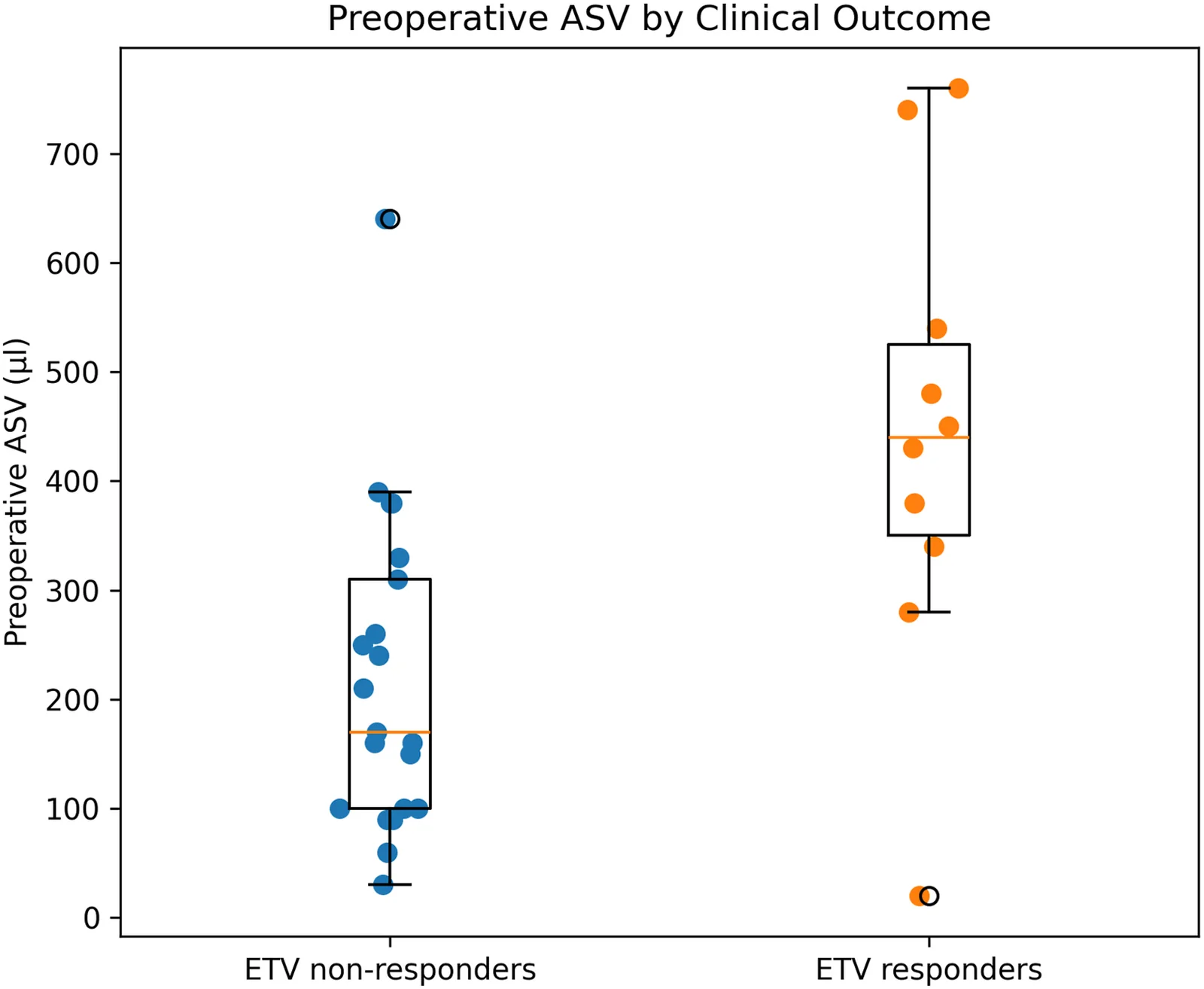
Preoperative ASV in ETV responders and non-responders. Dots represents individual patients; boxes show the median and interquartile range.

**Fig. 3:**
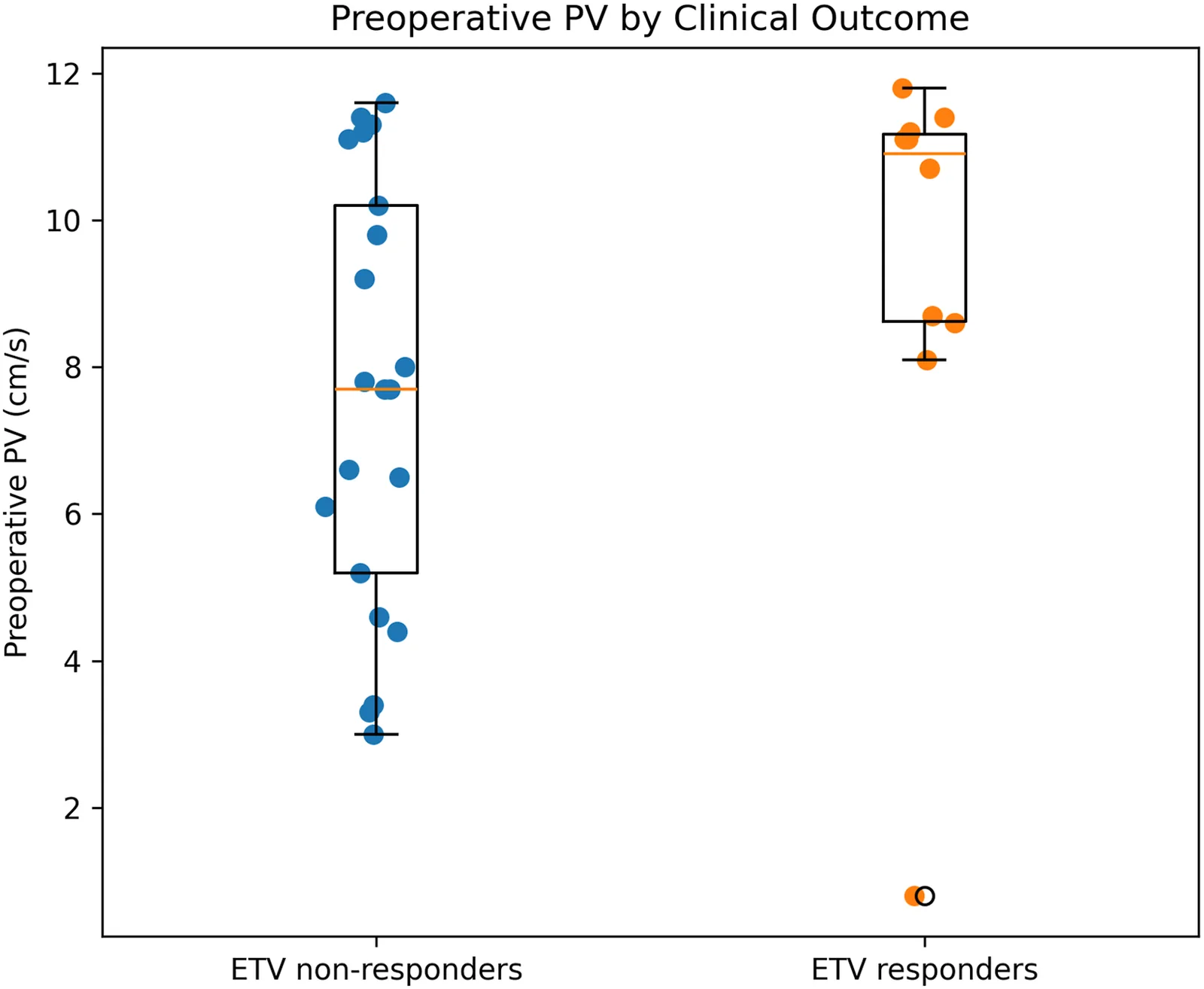
Preoperative PV in ETV responders and non-responders. Dots represents idividual; patients boxes show the median and interquartile range.

A significant difference was also observed in preoperative Kiefer score (p = 0.042), with lower scores associated with favorable clinical response. Among individual Kiefer subcomponents, only the cognitive domain (M) reached statistical significance, whereas the remaining components (G, I, H, D) did not demonstrate significant associations.

In univariable logistic regression analysis, preoperative ASV was significantly associated with sustained clinical response (odds ratio [OR] = 1.012 per 1 μl increase; 95% confidence interval [CI]: 1.001–1.023; p = 0.027). Other evaluated variables (PV, Net ASV, age, sex, NPH Radscale, presence of DESH) did not reach statistical significance.

In exploratory multivariable logistic regression including ASV, PV, total Kiefer score, and the cognitive subcomponent (M), preoperative ASV remained the only variable independently associated with sustained clinical response (p = 0.027). Spearman correlation analysis demonstrated the strongest correlation between clinical response and preoperative ASV (ρ = 0.44), followed by net ASV (ρ = 0.42), whereas correlation with preoperative PV was weaker (ρ = 0.32).

ROC analysis for preoperative ASV demonstrated good discriminatory capacity (area under the curve [AUC] = 0.82). A cut-off value of 280 μl yielded a sensitivity of 90%, specificity of 71%, positive predictive value of 60%, and negative predictive value of 94%.

Preoperative PV demonstrated lower discriminatory performance (AUC = 0.70). At a threshold of 8.1 cm/s, sensitivity was 90%, specificity 62%, positive predictive value 53%, and negative predictive value 93% (Fig. 4).

**Fig. 4:**
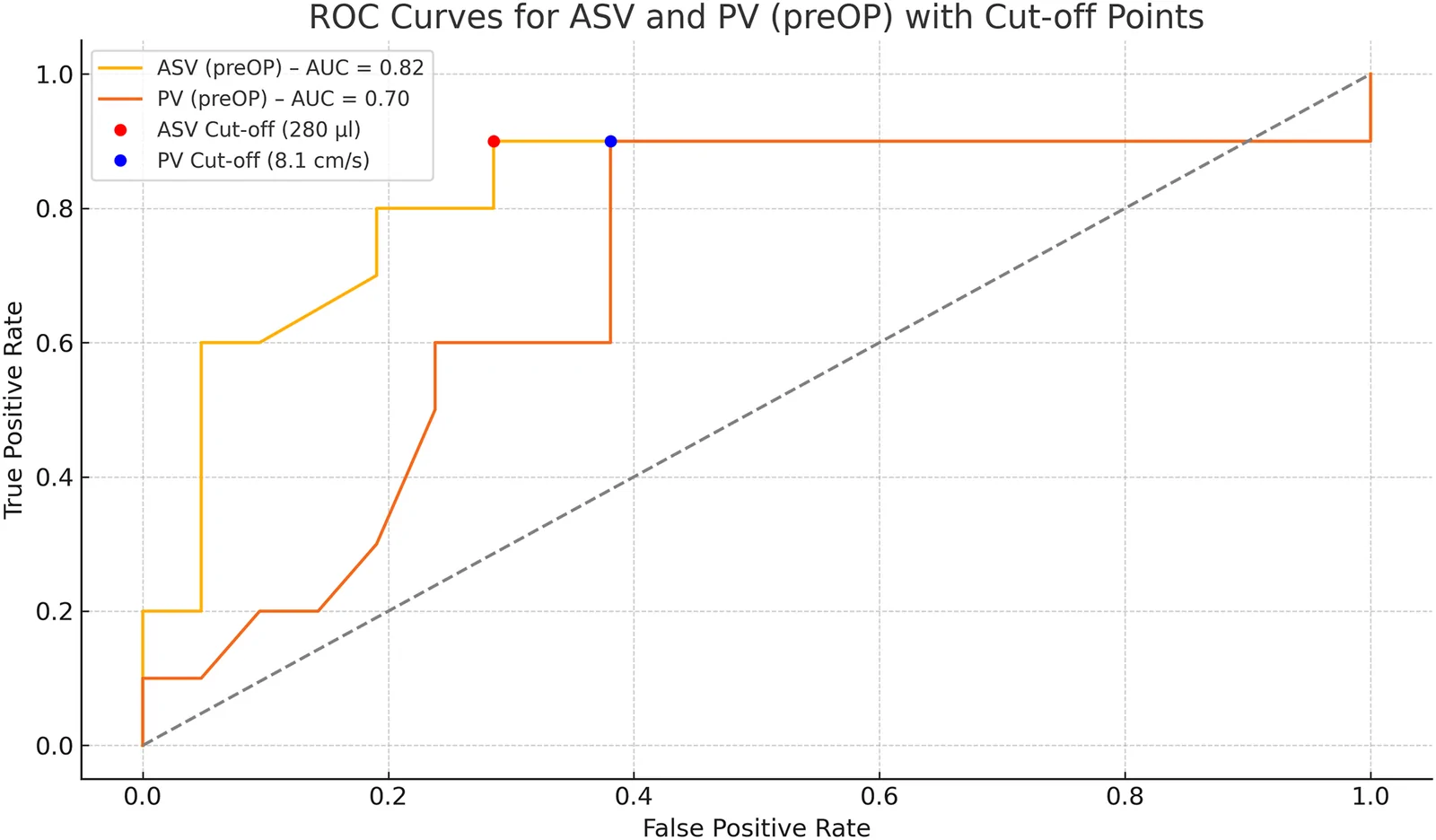
ROC curves for preoperative ASV and preoperative PV in discriminating sustained clinical response after ETV. AUC were 0.82 and 0.70, respectively; exploratory cut-offs were 280 μl and 8.1 cm/s.

Of the 21 patients in whom ETV was ineffective (Effect = 0), 20 subsequently underwent VP shunt implantation. One patient did not undergo shunt implantation due to advanced oncological disease. Clinical improvement following VP shunt implantation (Effect VP = 1) was observed in 16 patients (80%).

Within this subgroup, a significant positive correlation was found between the preoperative severity of continence impairment and clinical improvement after VP shunt implantation (ρ = 0.559; p = 0.010). Other evaluated variables (age, sex, PV preOP, ASV preOP, Net ASV preOP) were not significantly associated with clinical response to secondary shunt treatment.

Post hoc exploratory analysis evaluating clinical improvement following any surgical treatment (ETV or subsequent VP shunt implantation) did not demonstrate statistically significant associations between this combined clinical outcome and NPH Radscale, presence of DESH, PV (preOP), or ASV (preOP). In the combined treatment analysis, ASV (preOP) and PV (preOP) demonstrated weaker and statistically non-significant associations with positive clinical response compared to the analysis restricted to ETV alone.

## 4 Discussion

This prospective observational study evaluated clinical outcomes after primary ETV in patients with iNPH and examined the association between preoperative PC-MRI-derived CSF flow parameters and subsequent clinical response. Sustained clinical improvement was achieved in approximately one third of patients in our cohort, representing a lower proportion than reported in some earlier studies, where ETV success rates in iNPH reached 65–80%. However, in the authors’ recent systematic review, reported ETV outcomes varied widely across studies, reflecting heterogeneity in diagnostic criteria, patient selection, outcome definitions, and follow-up duration (Pištěk et al., 2025). These differences may partly reflect variation in cohort selection and outcome assessment; limited sample sizes in earlier cohorts may also have resulted in imprecise estimates of ETV benefit. In the present study, the lower response rate may additionally reflect the requirement for sustained clinical benefit at both 3 and 12 months. Direct numerical comparison with earlier reports should therefore be made cautiously.

In the overall cohort, a statistically significant reduction in PV was observed following surgery, whereas ASV and Net ASV did not change significantly. Moreover, postoperative changes in these parameters did not differ significantly between patients with and without sustained clinical response. Thus, the observed reduction in PV did not appear to be a marker of clinical benefit in this cohort. This pattern is compatible with a predominantly local effect of ETV on ventricular hydrodynamics rather than full normalization of global CSF dynamics. In contrast, ASV may remain influenced by global factors, including intracranial and ventricular compliance, vascular pulsatility, and CSF resorption capacity. The present data cannot determine the mechanism underlying these postoperative changes.

By contrast, the principal finding of this study was the statistically significant association between higher ASV (preOP) values and favorable clinical outcome after ETV. ROC analysis demonstrated good discriminatory capacity of ASV (preOP), and an exploratory threshold of ≥ 280 μl was associated with high sensitivity (90%) and moderate specificity (71%). These findings suggest that particularly low ASV (preOP) values may identify patients with a low likelihood of clinical benefit from ETV, which may have implications for patient selection in primary endoscopic treatment. In contrast, PV (preOP) demonstrated lower discriminatory performance and may have a complementary rather than decisive role in risk stratification.

ASV is a PC-MRI-derived measure of cardiac cycle-related aqueductal CSF pulsatility. Although increased ASV has been studied in iNPH as a possible marker of altered CSF dynamics and of response to CSF diversion, particularly VP shunting, its clinical utility remains uncertain. Reported ASV values and proposed thresholds vary substantially across studies, partly because of differences in PC-MRI acquisition, velocity-encoding settings, region-of-interest (ROI) placement, and post-processing (Whitley et al., 2024; Bradley, 2015; Luetmer et al., 2002; Scollato et al., 2008). Data specifically relating ASV to outcome after ETV remain sparse. Earlier ETV studies have more often evaluated PV or evidence of functional aqueductal stenosis (Kang et al., 2018). ASV should therefore be considered one component of a multimodal preoperative assessment rather than a stand-alone treatment-selection marker.

Accordingly, the association observed in this cohort should not be interpreted as a definitive predictor of ETV success. Higher ASV (preOP) values may represent relatively preserved brain compliance and potentially reversible stages of the disease. Conversely, lower CSF flow parameters may be associated with advanced parenchymal degeneration, in which the probability of clinical improvement following endoscopic intervention is limited (Bradley, 2015; Scollato et al., 2008). These observations further support the hypothesis that, from a hydrodynamic perspective, iNPH is not merely a disorder of CSF resorption but rather a complex disturbance of CSF dynamics.

From a pathophysiological standpoint, ETV may function as a partial bypass, modifying the transmission of pulsatile forces between the ventricular system and basal cisterns rather than directly normalizing global CSF pulsatility. In patients with relatively preserved parenchymal compliance and potentially reversible CSF dynamic disturbances, this altered hydrodynamic communication may be sufficient to reduce local intraventricular pulsatile stress and contribute to clinical improvement (Prusseit et al., 2009; Eshra, 2014; Gangemi et al., 2008; Greitz, 1993; Hailong et al., 2008; Jenkinson et al., 2009; Oliveira et al., 2015; Torsnes et al., 2014). However, in more advanced stages characterized by reduced parenchymal compliance or irreversible structural impairment, restoration of communication between the ventricular and subarachnoid compartments may be insufficient, and clinical improvement may require extracranial CSF diversion using a shunt system.

The absence of ETV-related surgical complications in our cohort supports the procedural safety of ETV when performed by experienced neurosurgeons. Furthermore, among patients who did not achieve sustained clinical response after ETV, secondary VP shunt implantation resulted in clinical improvement in 80% of cases. This suggests that primary ETV did not generally preclude subsequent benefit from standard shunt therapy.

However, this observation should not be interpreted as proof that an ETV-first strategy does not delay effective treatment, because the study lacked a randomized primary VP shunt control group and did not systematically assess interval clinical deterioration before secondary VP shunting.

Among clinical variables, total preoperative Kiefer score and particularly its cognitive subcomponent (M) were associated with clinical response to ETV, whereas other components, including gait disturbance (G) and continence impairment (I), did not reach statistical significance. This finding is consistent with previous observations suggesting that advanced cognitive deterioration reflects more severe and often irreversible brain damage.

### 4.1 Study limitations

Several limitations should be acknowledged. First, the study was limited by the small sample size (n = 31) and the low number of sustained responders (n = 10), which reduced statistical power and limited the robustness of multivariable modeling. Given the small cohort and multiple comparisons, both false-negative and false-positive findings cannot be excluded. Therefore, the regression and ROC analyses should be considered exploratory and hypothesis-generating.

Second, treatment allocation was non-randomized. Although patients were primarily considered for ETV, some elected conventional VP shunt implantation after standardized counseling. As the present analysis included only patients treated with primary ETV, direct comparison with immediate primary VP shunt implantation was not possible, and the potential effect of treatment delay cannot be determined.

Third, PC-MRI measurements may have been influenced by image quality, VENC settings, ROI placement, and vendor-specific post-processing. The ROC-derived ASV threshold of ≥ 280 μl was not externally validated and should not be interpreted as a clinically established decision threshold.

Finally, this was a single-center study performed by neurosurgeons experienced in neuroendoscopy.

Because the cohort comprised selected patients with iNPH who fulfilled the stated diagnostic criteria and chose primary ETV, the results may not generalize to unselected iNPH populations, patients selected for immediate VP shunting, or centres using different imaging or surgical practice.

## 5 Conclusion

This prospective single-center observational study suggests that primary ETV may lead to sustained clinical improvement in a selected subgroup of patients with iNPH. Higher ASV (preOP) measured by PC-MRI was associated with a more favorable clinical response after ETV, whereas preoperative PV showed weaker discriminatory performance.

These findings suggest that hyperdynamic aqueductal CSF flow may identify patients with a higher likelihood of response to ETV, possibly reflecting more preserved cerebral compliance and residual reversibility of the disease. However, the ASV threshold of ≥ 280 μl should be regarded as exploratory and hypothesis-generating rather than as an established clinical decision threshold.

Given the limited sample size, non-randomized design, and lack of external validation, ETV cannot be recommended as a standard treatment modality for iNPH based on the present data. Nevertheless, in carefully selected patients, it may represent a potential therapeutic alternative. Larger, ideally multicenter randomized studies are required to validate the role of PC-MRI-derived CSF flow parameters in guiding treatment decisions in iNPH.

## Ethics declaration

Written informed consent to take part in the study and to publish the article has been obtained from all participants or their legal representatives. The privacy rights of participants have been observed.

This study was performed in compliance with relevant laws, regulatory frameworks and guidelines where the research took place. This study was approved by the Ethics Committee of Krajská zdravotní, a.s., Masaryk Hospital, o.z. (Approval No. 292/30)

## Funding

This work was supported by an internal grant for scientific research of Krajská zdravotní a.s. (IGA-KZ-2025-1-12).

## Competing interests

The authors have no relevant financial or non-financial interests to disclose.
